# Prognostic value of the platelet-to-lymphocyte ratio for recurrent hemoptysis following bronchial artery embolization in patients with ronchiectasis

**DOI:** 10.1097/MD.0000000000050299

**Published:** 2026-08-21

**Authors:** Yuxin Duan, Weifan Sui, Zefeng Cai, Yimao Xia, Jianyun Li, Jianhua Fu

**Affiliations:** a Department of Interventional Radiology, The Affiliated People’s Hospital of Jiangsu University, Zhenjiang, Jiangsu, China.

**Keywords:** bronchial artery embolization, bronchiectasis, hemoptysis recurrence, platelet-to-lymphocyte ratio, prognostic model

## Abstract

Hemoptysis is a potentially life-threatening complication commonly observed in patients with bronchiectasis, often necessitating bronchial artery embolization (BAE). However, recurrence following BAE remains a significant clinical challenge. The platelet-to-lymphocyte ratio (PLR), a readily available inflammatory marker, has shown prognostic value in various pulmonary diseases. This study aimed to investigate the association between PLR and hemoptysis recurrence in patients with bronchiectasis who underwent BAE and to develop a prognostic model based on PLR. This retrospective study included 127 patients with bronchiectasis-related hemoptysis who underwent BAE between January 2018 and April 2022. Clinical, laboratory, and imaging data were collected. Restricted cubic spline analysis was used to evaluate the relationship between PLR and recurrence. Kaplan–Meier analysis assessed recurrence-free survival between high and low PLR groups, based on a cutoff determined by receiver operating characteristic analysis. Feature selection was performed using Boruta, LASSO regression, and random forest algorithms. A prediction model was constructed using intersecting variables and validated by time-dependent receiver operating characteristic, C-index, calibration, and decision curve analysis. PLR was significantly associated with hemoptysis recurrence, with a dynamic relationship observed over time: a linear association in the early follow-up period became nonlinear thereafter (*P* for overall <.05). Subgroup analyses confirmed the robustness of the association, with sputum culture status identified as a significant effect modifier. A cutoff PLR value of 123.3 was established, with higher levels indicating increased recurrence risk hazard ratio = 2.91, 95% Confidence interval CI: 1.61–5.29; *P* < .001). PLR was consistently identified as a top predictive feature across all 3 selection methods. The resulting prognostic model demonstrated relatively satisfactory discrimination and calibration, with area under the curve (AUC) values of 0.678, 0.766, and 0.776 at 1-, 3-, and 5-year follow-ups, respectively. PLR is an important and reliable predictor of hemoptysis recurrence following BAE in patients with bronchiectasis. The PLR-based model offers a practical tool for early risk stratification and may aid in clinical decision-making. Further multicenter prospective studies are warranted to validate these findings.

## 1. Introduction

Hemoptysis, a potentially life-threatening symptom, is associated with a variety of pulmonary diseases. Its clinical management remains challenging due to the dual risks of massive bleeding and airway obstruction. Among its many causes, bronchiectasis is one of the most common etiologies. In this condition, repeated infections lead to progressive structural damage of the airways, ultimately resulting in bleeding episodes. Hemoptysis has been reported in approximately 23% to 52% of patients with bronchiectasis.^[1–5]^ In China, the burden is particularly significant, with up to 70% of bronchiectasis patients experiencing hemoptysis,^[6]^ placing substantial strain on healthcare resources. Bronchial artery embolization (BAE) is widely recognized as an effective intervention for managing hemoptysis. However, recurrence after BAE remains a major clinical challenge, with reported rates ranging from 10% to 57%.^[7–9]^

The platelet-to-lymphocyte ratio (PLR) is an emerging composite biomarker that reflects both systemic inflammation and thrombotic activity. It has demonstrated notable diagnostic and prognostic value across a range of pathological conditions. In pulmonary medicine, prior studies have highlighted the predictive role of PLR in chronic obstructive pulmonary disease, lung cancer, and pulmonary embolism. Despite its established utility in multiple respiratory disorders,^[10–12]^ and recent preliminary studies have begun to explore inflammatory markers in the context of hemoptysis recurrence after BAE; ^[13]^ the specific association between PLR and bronchiectasis-related hemoptysis recurrence following BAE has not been fully elucidated. This knowledge gap is particularly relevant because PLR captures both inflammatory and thrombotic processes: key contributors to the pathophysiology of bronchiectasis-related hemoptysis.

In this retrospective study, we hypothesized that PLR may serve as a good predictor of hemoptysis recurrence after BAE in patients with bronchiectasis. If validated, PLR could offer a simple, cost-effective tool for risk stratification and support clinical decision-making in this high-risk population.

## 2. Methods

This retrospective study was approved by the Institutional Ethics Committee of the Affiliated People’s Hospital of Jiangsu University (Approval No. SQK-2025055-W), with a waiver of informed consent. All BAE procedures were performed in accordance with the standards of the Society of Interventional Radiology. The study was conducted in compliance with institutional and national ethical guidelines and adhered to the principles of the Declaration of Helsinki (1964) and its later amendments or comparable ethical standards.

### 2.1. Study participants

**Figure 1.**
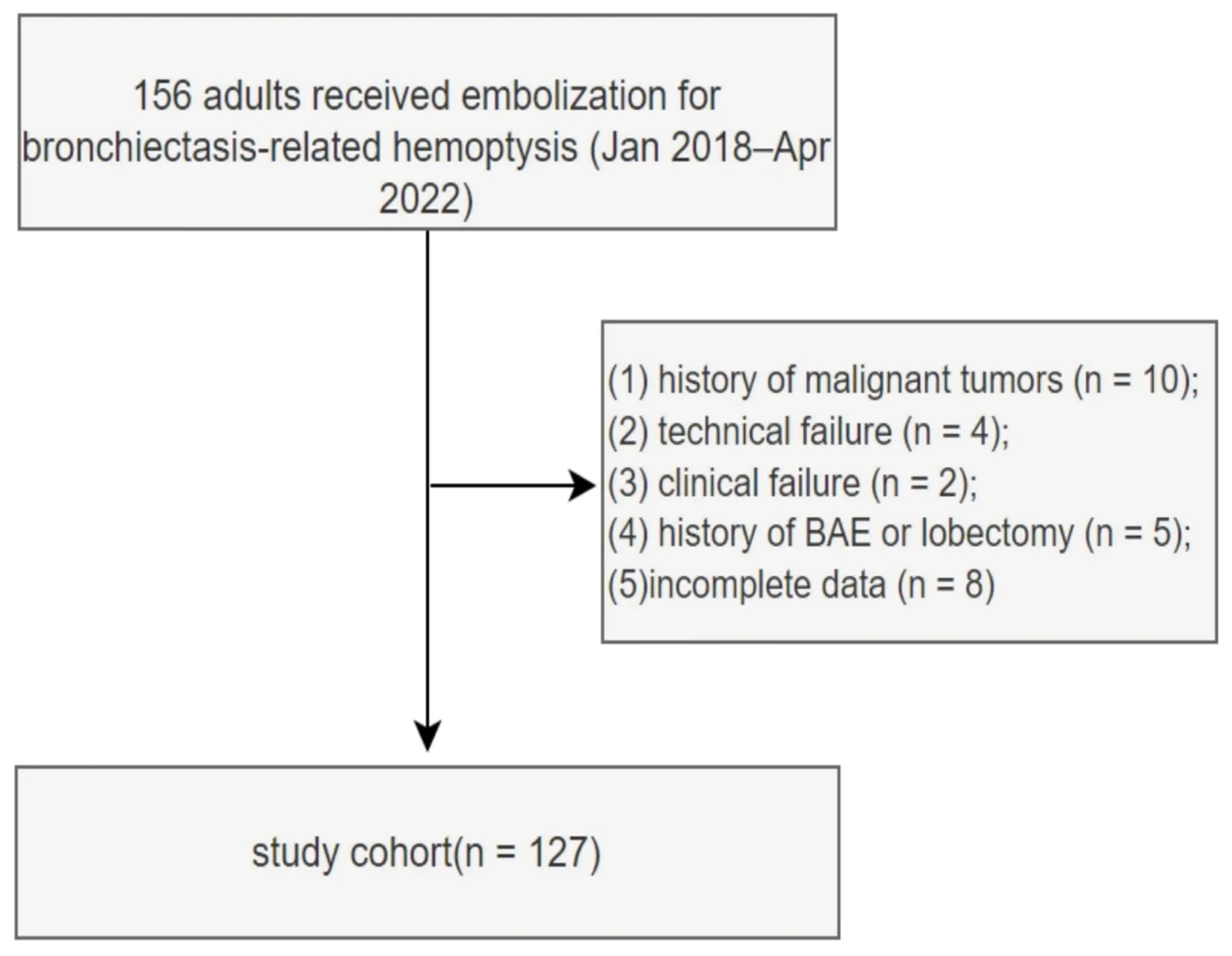
Flowchart illustrating the selection procedure for bronchiectasis patients with hemoptysis treated with bronchial artery embolization.

A total of 156 adult patients who underwent BAE for bronchiectasis-related hemoptysis at our institution between January 2018 and April 2022 were enrolled. Baseline characteristics, preprocedural laboratory results, chest computed tomography (CT), bronchial artery CT angiography and angiographic data were retrospectively collected. Patients were excluded if they had a history of malignancy (n = 10), experienced technical failure of embolization (n = 4) or clinical failure (n = 2), had a prior history of bronchial artery embolization or lobectomy (n = 5), or had incomplete clinical data (n = 8). After applying these inclusion and exclusion criteria, 127 patients were finally included in this analysis, and the patient selection flowchart is shown in Figure 1.

### 2.2. Data collection

The dataset included clinical characteristics, preprocedural peripheral blood test results, and imaging findings. Hemoptysis severity was classified based on daily blood loss as mild (≤100 mL), moderate (100–300 mL), or severe (>300 mL), according to previously established criteria.^[4]^ Peripheral blood samples were collected prior to embolization. A positive sputum culture was defined as the identification of any pathogenic microorganism in sputum samples obtained before BAE, in accordance with standard microbiological procedures.^[14]^ Pleural thickening >3 mm on CT was considered pathological.^[15]^ A history of hemoptysis was defined as any episode occurring within 6 months prior to the current admission.

All patients underwent high-resolution chest CT and bronchial artery computed tomography angiography prior to BAE to assess pulmonary infections and identify potential culprit vessels for accurate intraoperative targeting. Indications and procedural techniques followed the guidelines of the Society of Interventional Radiology.^[16]^ All interventions were administered under local anesthesia through the right femoral artery approach. Initial assessment of the thoracic aortic branches was accomplished with a 5F pigtail catheter. For selective angiography of vessels suspected to be the culprit, encompassing both bronchial arteries and non-bronchial systemic arteries, a 5F Cobra 3 catheter or a 5F left gastric catheter was utilized. The identification of the responsible vessels was based on characteristic angiographic manifestations, such as vascular dilatation, tortuosity, hypervascularity, Systemic artery–pulmonary circulation shunts and active contrast extravasation. Subsequent embolization procedures were performed using 2.7F or 2.4F microcatheters (Terumo Medical Corp., Japan; Boston Scientific, USA). The selection of embolic materials was tailored according to the diameter of the target vessels, with 350 to 560 μm polyvinyl alcohol particles and gelatin sponge particles both sourced from Hangzhou Alicon Pharmaceutical Co., Ltd. (Zhejiang, China), being the primary choices.

### 2.3. Recurrence and follow-up

Clinical success was characterized by either the complete resolution of hemoptysis or its decline to a minimal volume (<10 mL per day) following BAE. In contrast, clinical failure encompassed cases where hemoptysis recurred, or death occurred due to hemorrhage within 24 hours post-procedure.^[17]^ From a technical standpoint, a procedure was deemed successful if all abnormal vessels identified pre- or intraoperatively were effectively embolized. Failure in this context referred to partial embolization or the inability to pinpoint the responsible arteries.^[17]^ Recurrence was defined by any of the following events after an initial period of clinical success: daily hemoptysis reaching or exceeding 30 mL, the requirement for additional BAE or surgical interventions such as lobectomy, or mortality attributable to rebleeding.^[15]^

### 2.4. Statistical analysis

Only patients with complete follow-up data were included in the final analysis. Continuous variables were summarized as means ± standard deviations or medians with interquartile ranges (IQR), as appropriate. Categorical variables were presented as counts and percentages. Comparisons between categorical variables were performed using the *χ*^2^ test or Fisher’s exact test, as appropriate. Continuous variables were compared using the Student *t* test or the Wilcoxon rank-sum test, depending on data distribution. Restricted cubic spline (RCS) analysis was used to assess the nonlinear relationship between PLR and hemoptysis recurrence. Kaplan-Meier analysis was performed to compare recurrence-free survival between groups with high and low PLR levels. Additionally, subgroup analyses were conducted to evaluate whether potential confounders modified the association between PLR and recurrence. The Boruta algorithm, LASSO regression, and RF were used to select features based on their predictive value for hemoptysis recurrence. The variables identified by these methods were subsequently incorporated into the model construction. Time-dependent receiver operating characteristic (ROC) area under the curve (AUC) and C-index curves were used to evaluate the model’s discrimination and goodness of fit. Calibration curves were used to assess agreement between predicted and observed outcomes, while decision curve analysis (DCA) curves were performed to evaluate the model’s clinical utility. All statistical analyses were performed using R software (version 4.3.2, R Foundation for Statistical Computing, Vienna, Austria) for Windows. A two-sided *P* value of < .05 was considered statistically significant.

## 3. Results

### 3.1. Patient characteristics

In this retrospective analysis, 127 patients were enrolled and evaluated, with baseline characteristics summarized in Table 1. Over a mean follow-up period of 1246 days, the cohort was stratified into 2 groups based on hemoptysis recurrence: 73 patients (57.5%) remained recurrence-free, while 54 patients (42.5%) experienced recurrent hemoptysis. Demographic analysis showed a male predominance (n = 76, 59.8%), and 40 patients (31.5%) had a history of smoking. Intraoperative angiography revealed nonbronchial systemic arteries in 25 patients (19.7%) and Systemic artery–pulmonary circulation shunts in 49 patients (38.6%). No significant differences were found between groups in the prevalence of comorbidities, including diabetes mellitus, hypertension (HPN), and coronary heart disease. Microbiological evaluation identified positive sputum cultures in 41 patients (32.3%), with *P. aeruginosa* as the predominant pathogen (n = 25), followed by *K. pneumoniae* (n = 11) and *C. albicans* (n = 5).

**Table 1:** Baseline characteristics of the study patients.

Variable	Levels	Overall N = 127	Non-recurrence N = 73	Recurrence N = 54	*P*-value
Age, n (p%)					.064
	<60 yr	42.00 (33.07%)	29.00 (39.73%)	13.00 (24.07%)	
	>=60 yr	85.00 (66.93%)	44.00 (60.27%)	41.00 (75.93%)	
Gender, n (p%)					.114
	Female	51.00 (40.16%)	25.00 (34.25%)	26.00 (48.15%)	
	Male	76.00 (59.84%)	48.00 (65.75%)	28.00 (51.85%)	
Volume, mL, n (p%)					.286
	Massive	4.00 (3.15%)	1.00 (1.37%)	3.00 (5.56%)	
	Mild	94.00 (74.02%)	57.00 (78.08%)	37.00 (68.52%)	
	Moderate	29.00 (22.83%)	15.00 (20.55%)	14.00 (25.93%)	
Smoking, n (p%)					.007
	No	87.00 (68.50%)	43.00 (58.90%)	44.00 (81.48%)	
	Yes	40.00 (31.50%)	30.00 (41.10%)	10.00 (18.52%)	
Hemoptysis history, n (p%)					<.001
	No	72.00 (56.69%)	52.00 (71.23%)	20.00 (37.04%)	
	Yes	55.00 (43.31%)	21.00 (28.77%)	34.00 (62.96%)	
TOB, n (p%)					0.005
	Non-cystic	51.00 (40.16%)	37.00 (50.68%)	14.00 (25.93%)	
	Cystic	76.00 (59.84%)	36.00 (49.32%)	40.00 (74.07%)	
Pulmonary destruction, n (p%)					0.220
	Absent	121.00 (95.28%)	71.00 (97.26%)	50.00 (92.59%)	
	Present	6.00 (4.72%)	2.00 (2.74%)	4.00 (7.41%)	
HPN, n (p%)					.450
	No	92.00 (72.44%)	51.00 (69.86%)	41.00 (75.93%)	
	Yes	35.00 (27.56%)	22.00 (30.14%)	13.00 (24.07%)	
CHD, n (p%)					.904
	No	118.00 (92.91%)	68.00 (93.15%)	50.00 (92.59%)	
	Yes	9.00 (7.09%)	5.00 (6.85%)	4.00 (7.41%)	
Diabetes, n (p%)					.780
	No	114.00 (89.76%)	66.00 (90.41%)	48.00 (88.89%)	
	Yes	13.00 (10.24%)	7.00 (9.59%)	6.00 (11.11%)	
SPSs, n (p%)					.125
	Absent	78.00 (61.42%)	49.00 (67.12%)	29.00 (53.70%)	
	Present	49.00 (38.58%)	24.00 (32.88%)	25.00 (46.30%)	
NBSAs, n (p%)					.004
	Absent	102.00 (80.31%)	65.00 (89.04%)	37.00 (68.52%)	
	Present	25.00 (19.69%)	8.00 (10.96%)	17.00 (31.48%)	
Pleural thickening, n (p%)					.060
	Absent	15.00 (11.81%)	12.00 (16.44%)	3.00 (5.56%)	
	Present	112.00 (88.19%)	61.00 (83.56%)	51.00 (94.44%)	
Embolic materials, n (p%)					0.026
	GSP	21.00 (16.54%)	16.00 (21.92%)	5.00 (9.26%)	
	PVA	36.00 (28.35%)	24.00 (32.88%)	12.00 (22.22%)	
	PVA + GSP	70.00 (55.12%)	33.00 (45.21%)	37.00 (68.52%)	
Sputum culture, n (p%)					.004
	Negative	86.00 (67.72%)	57.00 (78.08%)	29.00 (53.70%)	
	Positive	41.00 (32.28%)	16.00 (21.92%)	25.00 (46.30%)	
DOC, mm, mean ± SD		3.30 ± 1.16	3.11 ± 1.10	3.57 ± 1.19	.028
NOB, median (IQR)		3.00 (2.00)	2.00 (1.00)	3.00 (3.00)	<.001
NOC, median (IQR)		2.00 (1.00)	2.00 (1.00)	3.00 (1.00)	.176
LOS, days, median (IQR)		9.00 (6.00)	9.00 (4.00)	11.00 (5.00)	.160
RBC, 10^9^/L, mean ± SD		4.11 ± 0.68	4.14 ± 0.71	4.07 ± 0.64	.540
HGB, g/L, mean ± SD		120.21 ± 20.82	123.14 ± 21.71	116.26 ± 19.03	.060
PLT, mean ± SD		171.51 ± 56.43	162.45 ± 57.75	183.76 ± 52.67	.033
Albumin, g/L, mean ± SD		37.21 ± 3.77	37.30 ± 3.93	37.09 ± 3.58	.746
TP, g/L, mean ± SD		64.52 ± 7.18	63.46 ± 6.87	65.94 ± 7.41	.058
PT, s, median (IQR)		11.30 (1.20)	11.30 (1.30)	11.40 (1.00)	.794
APTT, s, median (IQR)		27.60 (6.60)	27.70 (7.10)	27.00 (5.50)	.516
CRP, mg/L, median (IQR)		3.25 (7.78)	2.20 (5.48)	6.02 (10.26)	.836
WBC, 10^9^/L, median (IQR)		7.20 (4.00)	7.30 (4.20)	6.90 (4.00)	.610
ALC, 10^9^/L, median (IQR)		1.20 (0.88)	1.30 (1.00)	1.10 (0.70)	.011
NEUC, 10^9^/L, mean ± SD		5.82 ± 2.70	6.00 ± 2.83	5.58 ± 2.53	.385
NLR, median (IQR)		3.92 (4.98)	3.74 (5.35)	5.48 (4.45)	.687
PLR, median (IQR)		126.43 (108.69)	114.00 (90.00)	175.54 (144.44)	.009
SII, median (IQR)		628.33 (924.33)	588.00 (654.14)	889.00 (1082.44)	.481
CAR, median (IQR)		0.09 (0.24)	0.06 (0.14)	0.16 (0.28)	.967

ALC = absolute lymphocyte count, APTT = activated partial thromboplastin time, CAR = C-reactive protein to albumin ratio, CHD = chronic heart disease, CRP = C-reactive protein, DOC = diameter of culprit vessels, GSP = gelatin sponge particles PVAPolyvinyl alcohol, HGB = hemoglobin, HPN = hypertension, IQR = interquartile range, LOS = length of stay, NBSAs = non-bronchial systemic arteries, NEUC = neutrophil count, NLR = neutrophil-to-lymphocyte ratio, NOB = number of affected lobes in bronchiectasis, NOC = number of culprit arteries, PLR = platelet-to-lymphocyte ratio, PLT = platelet, PT = prothrombin time, RBC = red blood cell, SD = standard deviation, SII = systemic immune-inflammation index, SPSs = Systemic arterial-pulmonary circulation shunts, TOB = Type of bronchiectasis, TP = Total protein.

### 3.2. PLR levels and their relation to bronchiectasis-related hemoptysis recurrence

**Figure 2.**
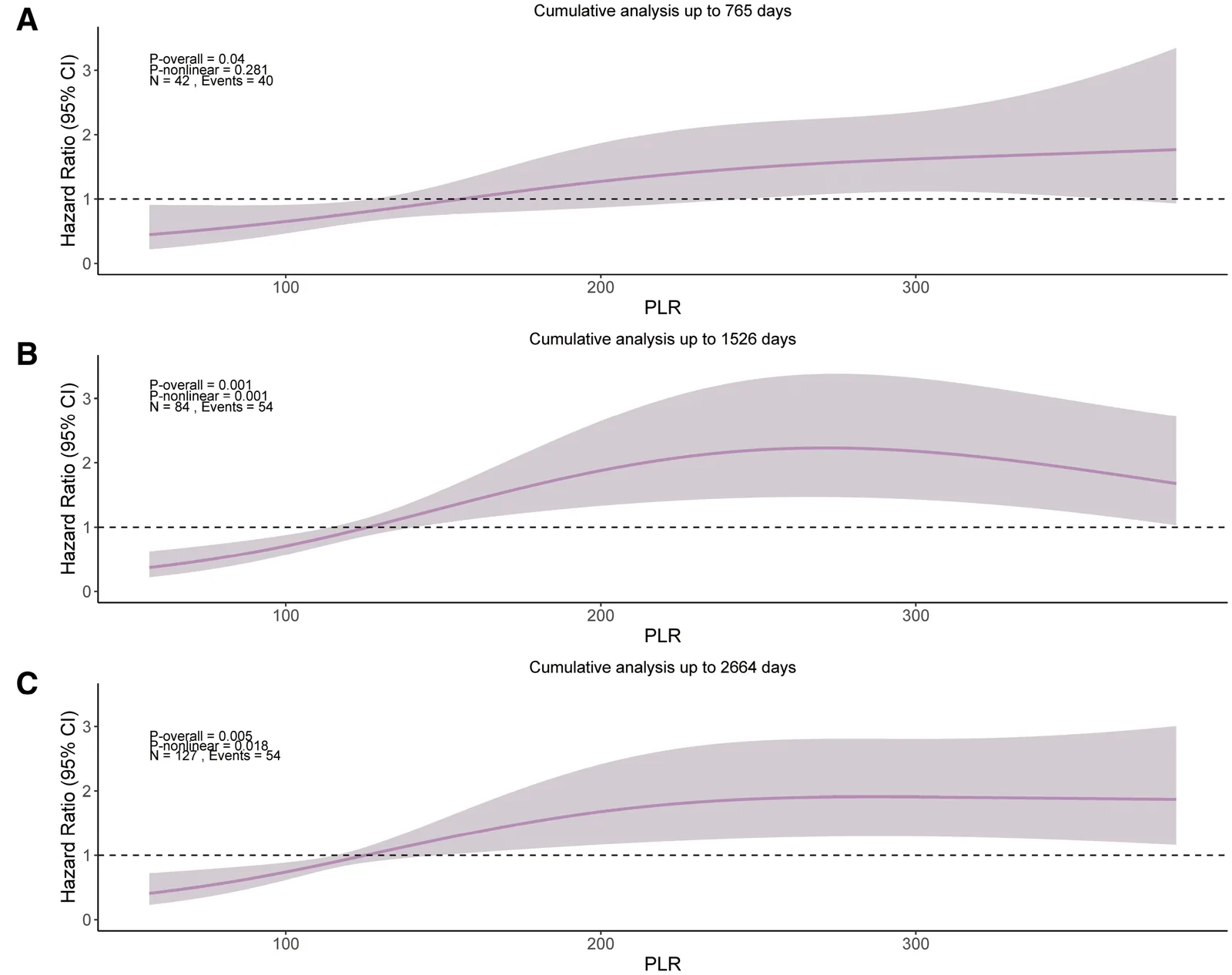
Restricted cubic spline analysis illustrating the association between the PLR and the risk of hemoptysis recurrence in patients with bronchiectasis. (A) RCS curve at follow-up of 765 days. (B) RCS curve at follow-up of 1526 days. (C) RCS curve at follow-up of 2664 days. CI = confidence interval, PLR = platelet-to-lymphocyte ratio, RCS = restricted cubic spline.

RCS analysis revealed a strong association between PLR levels and the risk of hemoptysis recurrence throughout the follow-up period (Fig. 2). The association between PLR and recurrence risk exhibited a temporal pattern. During the first third of the follow-up period, a linear relationship was observed between PLR levels and the probability of recurrence (Fig. 2A). As the follow-up progressed beyond this phase, the relationship became nonlinear (Fig. 2B and Figure 2C). The overall association remained statistically significant throughout the entire follow-up period (*P* for overall <.05). Multivariate Cox regression models were constructed to evaluate the association between PLR levels and the risk of hemoptysis recurrence in patients with bronchiectasis. In the unadjusted model (Crude Model), elevated PLR levels were significantly associated with an increased risk of recurrence (hazard ratio [HR] = 2.91, 95% confidence interval (Confidence interval [CI]: 1.21–5.29, *P* < .001). Model 1, which adjusted for age, gender, and hemoptysis volume, yielded similar results (HR = 2.89, 95% CI: 1.56–5.31, *P* < .001). In Model 2, additional adjustments were made for TOB, hypertension, embolic materials, coronary heart disease, diabetes, systemic immune-inflammation index, C-reactive protein to albumin ratio and neutrophil-to-lymphocyte ratio. Even after further adjustment, higher PLR levels remained significantly associated with an increased risk of recurrence. Detailed results are presented in Table 2.

**Table 2:** Cox proportional hazard ratios (HR) for bronchiectasis-related hemoptysis.

Varoable	HR* (95% CI)	*P* value
Crude model†	2.91 (1.21–5.29)	<.001
Model 1‡	2.89 (1.56–5.31)	<.001
Model 2§	4.50 (2.28–8.89)	<.001

CAR = C-reactive protein to albumin ratio, CHD = chronic heart disease, HPN = hypertension, NLR = neutrophil-to-lymphocyte ratio, PLR = platelet-to-lymphocyte ratio, SII = systemic immune-inflammation index, TOB = type of bronchiectasis.

* HR hazard ratio.

† Crude model: No covariates were adjusted.

‡ Model 1: Adjusted for Age, Gender, and Volume.

§ Model 2: Model 1 and adjusted for TOB, embolic materials, HPN, CHD, Diabetes, SII, CAR and NLR.

### 3.3. Survival analysis comparing high and low PLR levels for hemoptysis recurrence in bronchiectasis patients

To determine a clinically applicable threshold, ROC analysis was performed for PLR. The optimal cutoff value was identified as 123.3, yielding a sensitivity of 72.22% and a specificity of 61.64% for predicting hemoptysis recurrence in patients with bronchiectasis.

**Figure 3.**
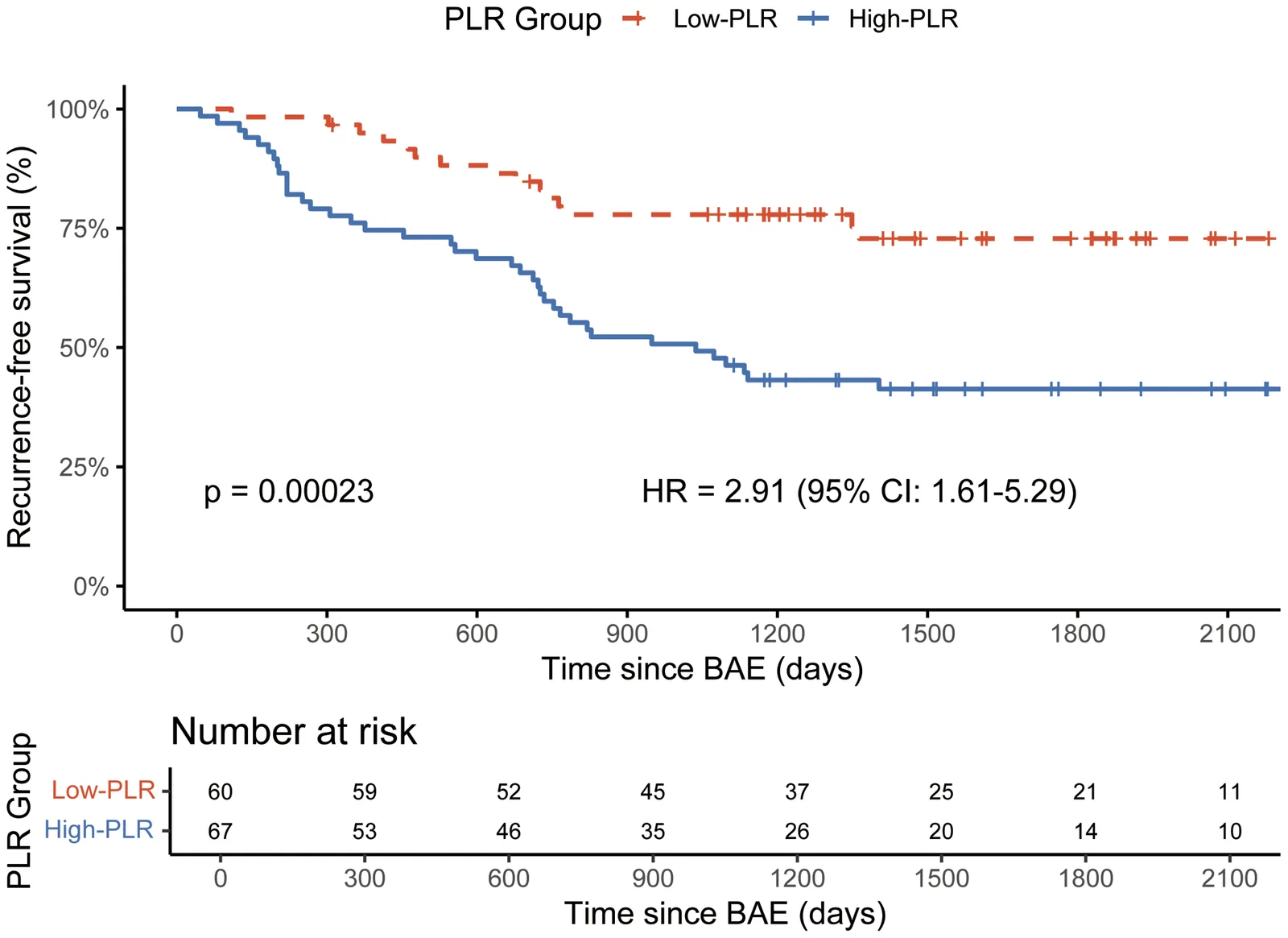
Kaplan–Meier survival curves comparing hemoptysis recurrence between high and low PLR groups based on the optimal cutoff value. BAE = bronchial artery embolization, HR = hazard ratio, PLR = platelet-to-lymphocyte ratio.

Based on this threshold, patients were stratified into low- and high-PLR subgroups for Kaplan-Meier survival analysis (Fig. 3). The survival curves demonstrated that patients in the high-PLR group had a significantly higher risk of bronchiectasis-related hemoptysis recurrence compared with those in the lower-PLR group (*P* < .001; HR = 2,91, 95% CI: 1.61–5.29).

### 3.4. Subgroup analysis

Subgroup analysis was performed to evaluate whether the prognostic value of PLR was modified by variables including gender, age, diabetes, hypertension, bronchiectasis type, smoking history, hemoptysis history, and sputum culture status.

**Figure 4.**
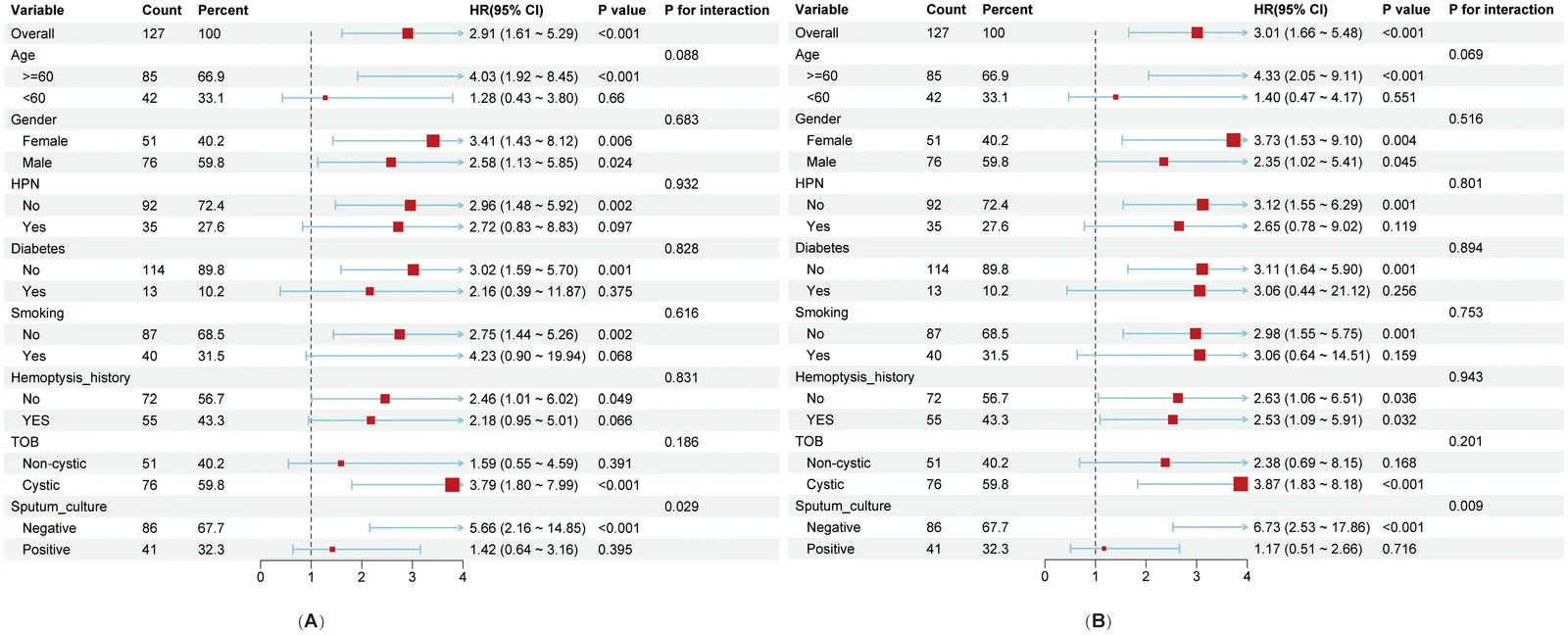
Subgroup analysis of the association between PLR and hemoptysis recurrence. (A) Unadjusted hazard ratios. (B) Adjusted hazard ratios. HPN = hypertension, HR = hazard ratio, PLR = platelet-to-lymphocyte ratio, TOB = type of bronchiectasis.

Across most subgroups, higher PLR levels were consistently associated with an increased risk of recurrence, with both unadjusted (Fig. 4A) and adjusted (Fig. 4B) HRs exceeding 1 and reaching statistical significance (*P* < .05). No significant interaction was observed between PLR and any of these variables (*P* for interaction > .05), except for sputum culture status, where a significant interaction was detected (*P* for interaction < .05). In this subgroup, the predictive association between PLR and recurrence remained statistically significant in both unadjusted and adjusted models.

### 3.5. Feature importance ranking for bronchiectasis-related recurrence prediction using Boruta, LASSO regression, and random forest algorithms

**Figure 5.**
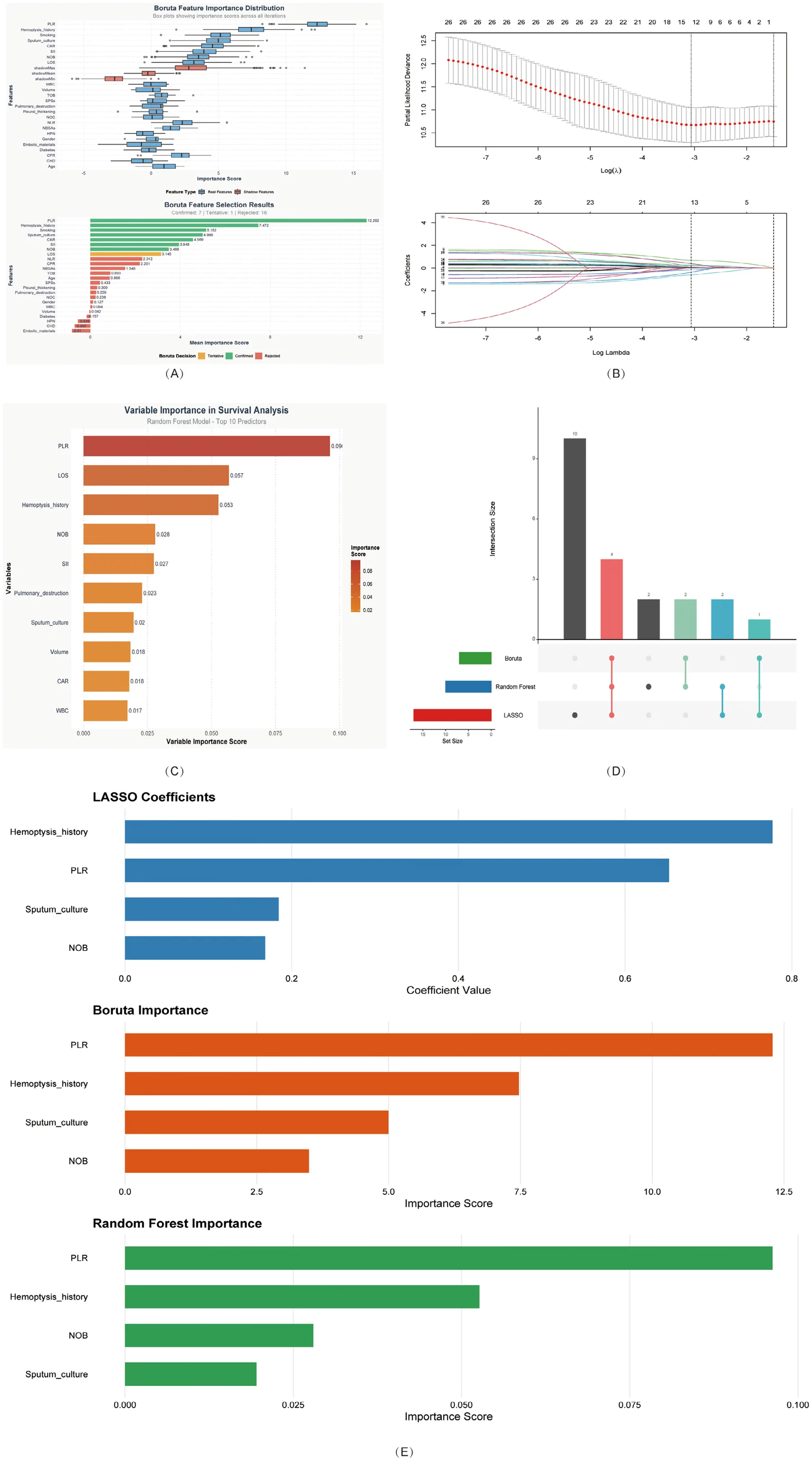
Feature selection for predictors of hemoptysis recurrence in patients with bronchiectasis. (A) Variable importance ranking generated by the Boruta algorithm. (B) LASSO regression with 10-fold cross-validation identified key predictors based on lambda.min. (C) Top 10 predictive variables selected by the random forest algorithm. (D) UpSet plot illustrating the intersection of selected variables across Boruta, LASSO, and RF methods. (E) Summary of PLR’s ranking across all 3 algorithms. CAR = c-reactive protein to albumin ratio, CHD =coronary heart disease, HPN = Hypertension, LOS = length of stay, NOB = number of affected lobes in bronchiectasis, NLR = neutrophil-to-lymphocyte ratio, NOC = number of culprit arteries, PLR = platelet-to-lymphocyte ratio, SII = systemic immune-inflammation index, SPSs = systemic arterial-pulmonary circulation shunts, TOB = type of bronchiectasis.

To identify the most predictive variables for hemoptysis recurrence in patients with bronchiectasis, 3 feature selection methods were employed: the Boruta algorithm (Fig. 5A), LASSO regression using the minimum lambda value (Fig. 5B), and random forest, selecting the top 10 ranked features (Fig. 5C). The final set of predictive variables was determined by the intersection of those identified by all 3 methods (Fig. 5D). Notably, PLR emerged as the most important variable in both the Boruta and random forest analyses, and as the second most important variable in the LASSO regression. This consistent ranking across methods highlights the relatively robust predictive value of PLR for hemoptysis recurrence (Fig. 5E).

### 3.6. Establishment and validation of the prediction model

**Figure 6.**
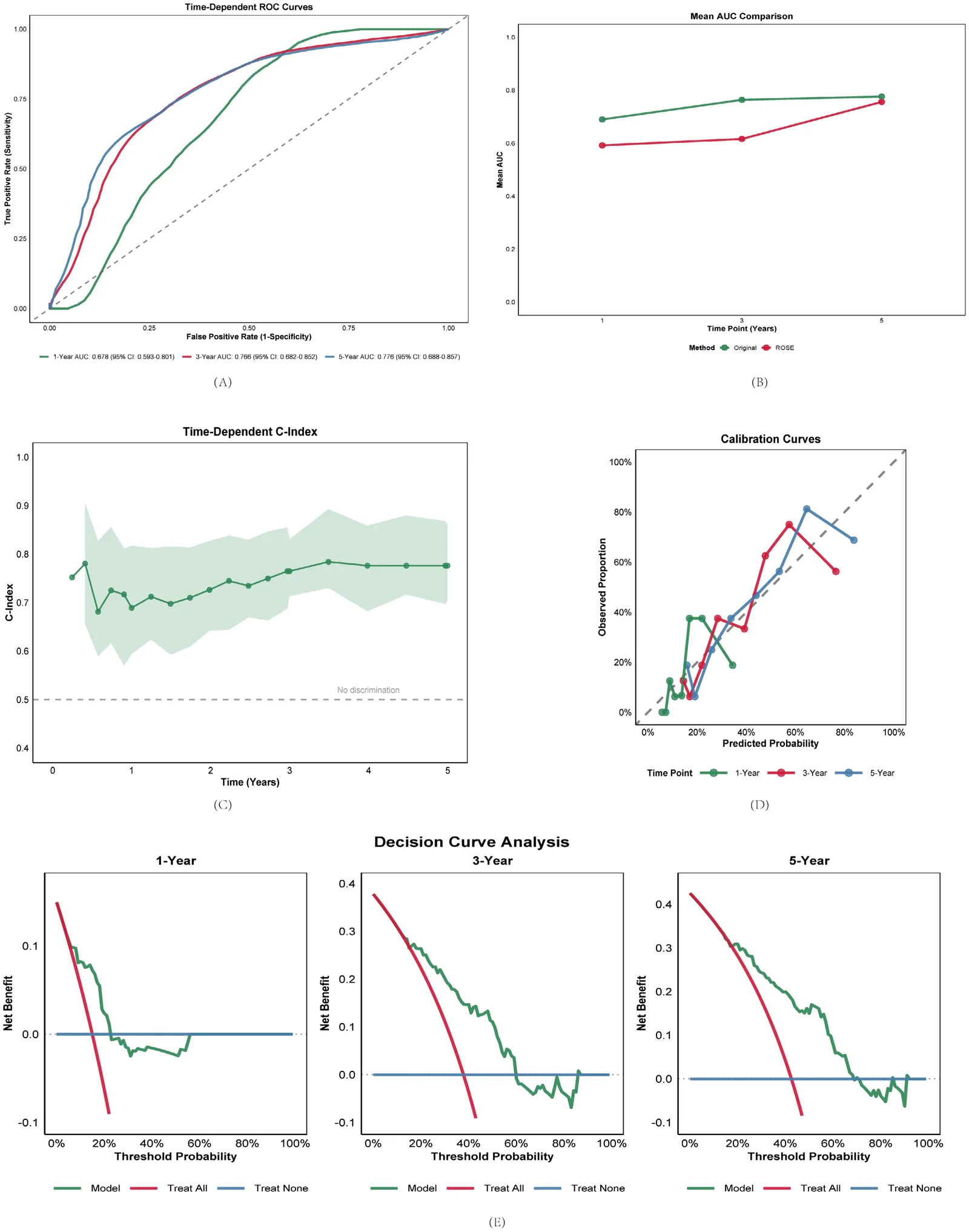
Performance evaluation of the recurrence prediction model based on the PLR. (A) Time-dependent ROC curves at 1, 3, and 5 years. (B) Dynamic AUC curve over the entire follow-up period. (C) Concordance index curve for overall model discrimination. (D) Calibration curve comparing predicted and observed recurrence probabilities. (E) DCA demonstrating the clinical net benefit of the model across a range of threshold probabilities. AUC = area under the curve, CI = confidence interval, DCA = decision curve analysis, ROC = receiver operating characteristic.

Given the limited sample size, the dataset was not split into separate training and validation cohorts. Instead, 50-fold repeated cross-validation was performed using 2 complementary approaches: the original method, which preserved the natural class distribution to reflect real-world clinical conditions; and the ROSE (Random Over-Sampling Examples) method,^[18]^ which applied kernel density estimation–based synthetic oversampling to address class imbalance. While the original approach preserved the inherent characteristics of the dataset, it remained vulnerable to the effects of class imbalance. In contrast, the ROSE method rebalanced the class distribution, thereby improving the model’s sensitivity in detecting minority class events. The model demonstrated good discriminatory ability, with AUCs of 0.678 (95% CI: 0.593–0.801), 0.766 (95% CI: 0.682–0.852), and 0.776 (95% CI: 0.688–0.857) at 1-, 3-, and 5-year follow-ups, respectively (Fig. 6A). The time-dependent AUC and C-index curves are shown in Figure 6B and Figure 6C. The calibration curve indicated good agreement between predicted and observed outcomes, reflecting good predictive accuracy (Fig. 6D). Additionally, DCA revealed a substantial net clinical benefit across the follow-up period, supporting the model’s clinical utility (Fig. 6E).

To rigorously evaluate the model’s generalizability and address concerns regarding overfitting in the context of a small sample size and high-dimensional parameters, we followed the bootstrap optimism framework proposed by Harrell et al.^[19]^ and supplemented it with data-perturbation stability analysis. After 1000-bootstrap optimism correction, the C-index remained virtually unchanged (0.703). Data-perturbation analysis (5 % Gaussian noise, 1000 runs) yielded a mean absolute ΔC-index of 0.0003 (95 % CI 0.0008–0.003), confirming minimal over-fitting despite the modest event size, as shown in Fig. S1, Supplemental Digital Content 1.

## 4. Discussion

In our previous preprint (Duan et al 2025), we investigated the prognostic impact of the computed tomography bronchiectasis score on hemoptysis outcomes following BAE. However, to incorporate factors such as composite inflammatory markers, this study expands on our earlier work by including relevant variables and employing various feature selection methods. This retrospective study examined the association between the PLR and hemoptysis recurrence in patients with bronchiectasis. The findings consistently demonstrated that elevated PLR is a significant prognostic factor, as validated through multiple statistical methods and analytical frameworks.

Conservative management, such as the use of anti-inflammatory and hemostatic agents, is commonly employed for patients presenting with mild to moderate hemoptysis.^[20]^ However, approximately half of the patients with bronchiectasis-related hemoptysis eventually require BAE.^[21]^ BAE is more effective for controlling acute hemorrhage than for addressing the underlying pathology of bronchiectasis. However, recurrence remains common even after technically successful embolization, largely due to the persistent airway inflammation characteristic of bronchiectasis. The high rates of hemoptysis recurrence and hospital readmission place a considerable economic burden on both families and the healthcare system. Consequently, establishing early prognostic tools and recognizing individuals at elevated risk of recurrence may help clinicians adopt more personalized and preemptive therapeutic approaches, thereby minimizing the likelihood of hemoptysis recurrence.

However, aside from CRP, the clinical use of many inflammatory biomarkers is limited by high costs and long turnaround times. In contrast, inflammatory indices such as the neutrophil-to-lymphocyte ratio and PLR, which can be readily calculated from routine hematological tests, have gained increasing attention in inflammatory diseases including bronchiectasis due to their rapid availability, low cost, and ease of interpretation. In earlier work, Lu et al demonstrated that an elevated PLR was associated with a higher risk of severe hemoptysis recurrence.^[13]^ This finding aligns with our conclusion that an increased PLR is linked to hemoptysis in patients with bronchiectasis, thereby reinforcing the reliability of our results. Within the pathophysiological context of bronchiectasis, chronic airway inflammation induces significant alterations in both platelet activation and lymphocyte dynamics, along with the sustained release of inflammatory mediators from these and other immune cells. This persistent inflammatory milieu contributes to progressive vascular injury and pathological angiogenesis within the bronchial walls. For example, activated platelets amplify inflammatory responses and increase vascular permeability through the release of potent mediators such as vascular endothelial growth factor (VEGF) and thromboxane A_2_ (TXA_2_),^[22]^ which may collectively predispose patients to erythrocyte extravasation and clinically manifesting hemoptysis.

RCS analysis demonstrated a time-dependent association between PLR and hemoptysis recurrence in patients with bronchiectasis. A linear relationship was observed during the first third of the follow-up period, which transitioned to a nonlinear pattern thereafter. The overall association remained statistically significant throughout the entire follow-up period (*P* < .05). This finding was further supported by subgroup analyses across potential modifying factors, confirming the robustness of the association. Notably, sputum culture status significantly modified the relationship between PLR and hemoptysis recurrence, with *P* for interaction <.05 both before and after adjustment. This suggests that the prognostic value of PLR may be influenced by the presence of airway infection. Previous studies have demonstrated that *P. aeruginosa*, a key pathogen in bronchiectasis,^[22,23]^ is associated with poorer clinical outcomes and a heightened inflammatory response in the airways. Mechanistically, *P. aeruginosa* secretes phospholipase C, which promotes the release of arachidonic acid metabolites, including thromboxane B_2,_ from immune cells.^[24,25]^ Although not all bacterial toxins elevate thromboxane levels, this pathway provides a biologically plausible link between *P. aeruginosa* infection and thromboxane A_2_–mediated platelet activation, vasoconstriction, and increased vascular permeability.^[26]^ In this context, elevated thromboxane levels may compromise pulmonary vascular integrity, thereby contributing to hemoptysis in patients with bronchiectasis. These findings carry important clinical implications, as the PLR can be readily calculated prior to bronchial artery embolization to facilitate early risk stratification and support more personalized and targeted treatment strategies. This result aligns with previous research in other pulmonary diseases, including COPD,^[27–29]^ pneumonia,^[30]^ and acute pulmonary embolism.^[31]^ These results further emphasize the importance of PLR in prognostic assessment for patients with bronchiectasis-related hemoptysis.

Another key finding was the PLR cutoff value of 123.3, determined by ROC analysis. PLR levels were significantly higher in the recurrence group. Kaplan–Meier analysis based on this threshold further supported its prognostic relevance. To assess the predictive value of PLR for hemoptysis recurrence, we applied 3 feature selection methods: Boruta, LASSO regression, and RF. All 3 methods consistently identified PLR as a relevant predictor. Moreover, compared with other inflammatory markers, PLR demonstrated the strongest predictive performance, as shown in Fig. S2, Supplemental Digital Content 2. A prediction model incorporating PLR was subsequently developed using the overlapping variables identified by the 3 methods. The model demonstrated satisfactory predictive performance, as shown by time-dependent ROC, AUC, and C-index curves. Integrated Brier scores remained below 0.25 throughout the follow-up period (Fig. S3, Supplemental Digital Content 3). The calibration curve indicated good agreement between predicted and observed outcomes. DCA showed that the model yielded a higher net benefit than individual predictors across a range of threshold probabilities. Finally, this PLR-based prognostic model may serve as a practical and intuitive tool to assist clinicians in making informed treatment decisions for patients with bronchiectasis-related hemoptysis.

This study has several limitations. First, as a single-center retrospective study, it is subject to inherent selection bias, which may have affected the accuracy and generalizability of the findings. Second, the sample size was limited, and the dataset was not split into training and testing sets; instead, internal validation was performed. In future research, the use of larger datasets and external validation could more robustly demonstrate the predictive value of PLR for post-BAE outcomes in patients with bronchiectasis-related hemoptysis. Third, while PLR alone demonstrated good predictive value, integrating it with additional inflammatory markers may further improve the accuracy of recurrence prediction. Future studies should explore multi-marker models to optimize risk stratification in clinical practice. Furthermore, in light of ongoing predictive modeling reliant on CT imaging, the assimilation of these hematological variables with imaging-derived parameters has the potential to substantially reinforce the robustness of the model and elevate its translational relevance.

## 5. Conclusion

Our study demonstrates that PLR is a strong prognostic indicator for hemoptysis recurrence in patients with bronchiectasis following BAE. The PLR-based predictive model provides a practical and efficient tool to assist clinicians in early risk stratification and individualized therapeutic planning. Nevertheless, multicenter prospective studies are warranted to further validate its predictive utility and generalizability.

## Author contributions

**Funding acquisition:** Yuxin Duan.

**Validation:** Zefeng Cai.

**Software:** Yimao Xia.

**Supervision:** Jianyun Li.

**Writing – original draft:** Yuxin Duan.

**Writing – review & editing:** Yuxin Duan, Weifan Sui, Jianhua Fu.






