# Supplementary material for: Prognostic value of the platelet-to-lymphocyte ratio for recurrent hemoptysis following bronchial artery embolization in patients with ronchiectasis
Source: Medicine (Baltimore). 2026 Aug 21;105(34):e50299. doi: 10.1097/MD.0000000000050299 (PMC13502753; doi:10.1097/MD.0000000000050299)
Supplement: Supplementary file 1 [file medi-105-e50299-s001.docx]

**Fig.S1 Distribution of C-index across 1,000 bootstrap samples after adding 5 % Gaussian noise to PLR; red dashed line indicates the original C-index value.**
